# Women’s views and experiences of a mobile phone-based intervention to support post-abortion contraception in Cambodia

**DOI:** 10.1186/s12978-017-0329-y

**Published:** 2017-06-12

**Authors:** Chris Smith, Sokhey Ly, Vannak Uk, Ruby Warnock, Caroline Free

**Affiliations:** 1 Department of Population Health, London School of Hygiene and Tropical Medicine, Room 150, Keppel Street, London, WC1E 7HT UK; 2 Marie Stopes International, Phnom Penh, Cambodia; 3 Department of Obstetrics, Gynecology and Reproductive Sciences, University of California, San Francisco, USA

## Abstract

**Background:**

The MObile Technology for Improved Family Planning (MOTIF) trial assessed a mobile phone-based intervention comprising voice messages and counsellor support to increase post-abortion contraception at four Marie Stopes International clinics in Cambodia. The aim of this process evaluation was to assess women’s views and experiences of receiving the MOTIF intervention, gain insights into the mechanism of action of the intervention and seek recommendations for improvements.

**Methods:**

We conducted a qualitative study comprising15 semi-structured interviews with women who had received the intervention and undertook a simple thematic analysis.

**Results:**

We identified themes relating to communication via mobile phone, supporting contraception use, broader post-abortion care, interaction with family and friends and suggestions for improvement. The majority of women were positive about the mobile phone-based intervention to support contraception use and reported it to be a convenient way to ask questions or get advice without going to a health centre, although a few women found the voice messages intrusive.

The intervention supported contraception use by provision of information, encouragement, reminders to return to clinic, reassurance and advice for problems and had a positive effect on contraceptive uptake and continuation. Women reported a sense of being cared for and received support for additional physical and emotional issues. Most women thought that the duration of the intervention and frequency of messages were acceptable.

**Conclusions:**

The majority of women were positive about the mobile phone-based intervention which provided support for contraception use as well as additional physical and emotional issues. The study provides some insights into how the intervention might have worked and considers how the intervention could be improved.

## Plain English summary

Our MObile Technology for Improved Family Planning (MOTIF) trial assessed a mobile phone-based intervention comprising six interactive voice messages +/− counsellor support to increase contraception use amongst women seeking abortion services at four Marie Stopes International clinics in Cambodia. The aim of this study was to explore women’s views and experiences of receiving the MOTIF intervention, gain further insights into how the intervention worked, and seek recommendations for improvements.

We conducted 15 interviews with women who had received the intervention. We recorded and analysed the interview transcripts to identify the main themes. We found that the majority of women were positive about the mobile phone-based intervention to support contraception use and reported it to be a convenient way to ask questions or get advice in confidence without going to a health centre, although a few women found the voice messages intrusive. Women reported that the intervention, in particular phone counselling, supported contraception use by provision of information, encouragement, reminders to return to clinic, reassurance and advice for problems. Women reported a sense of being cared for and received support for additional physical and emotional issues. Most women thought that the duration of the intervention and frequency of messages were acceptable.

In conclusion, the majority of women were positive about the mobile phone-based intervention which provided support for contraception use as well as additional physical and emotional issues. The study provides some insights into how the interventions might have works and considers how the intervention could be improved.

## Background

In recent years several randomised controlled trials have assessed interventions delivered by mobile phone (‘mHealth’) to improve contraception use [1]. Interventions have sought to improve adherence to specific methods of contraception such as oral contraceptive or injectable [2–4], or improve use of a range of contraceptive methods [5–7]. To date, the trial evidence for mobile phone-based interventions to increase contraception use is mixed. A mixture of uni-directional and interactive daily educational text messages improved oral contraceptive adherence at 6 months in the USA [2]. In our MObile Technology for Improved Family Planning (MOTIF) trial, a series of voice messages with additional counsellor support improved use of effective post-abortion contraceptive in Cambodia at four but not 12 months [5]. Automated text message reminders did not improve oral contraceptive or injectable adherence in two small trials in the USA [3, 4]. Text messages and role model stories were reported to be associated with increased knowledge but not behaviour change in Kenya [7]. Thus, whilst interventions delivered by mobile phone to improve contraception use show promise, greater understanding of what works, for whom and under what circumstances is required. Process evaluation alongside randomised controlled trials can enhance understanding of why a certain intervention works or does not work [8, 9].

The study reports women’s views and experiences of receiving voice messages and counsellor support for post-abortion contraception in the MOTIF trial. The MOTIF trial protocol, results and description of the intervention development are reported elsewhere [5, 10, 11]. In brief, the intervention comprised six automated interactive voice messages over the three-month post-abortion period. Women could press ‘1’ to request to speak to a counsellor, press ‘2’ if they did not require a call back, or press ‘3’ to opt-out of receiving further messages. Phone counselling aimed to increase contraception use by providing information about a range of methods and advice for side-effects. If the woman requested, the counsellor could also discuss contraception with the husband or partner. Clients that chose to receive the oral contraceptive or injectable could opt to receive additional reminder messages appropriate to their method.

Amongst 249 women that received the intervention, around half actively requested to speak to a counsellor (pressed ‘1’) and over 90% spoke to a counsellor at some stage. Women who spoke to the counsellor having requested to (by pressing ‘1’) were more likely to be using effective contraception at 4 months compared to women who didn’t request or speak to the counsellor (Odds Ratio 4.39; 95% CI: 1.15–16.71). Increased parity, a history of >2 previous induced abortions, lower socio-economic status, and medical abortion were associated with requesting to speak to a counsellor [12]. This study aims to explore women’s views and experiences of receiving the MOTIF intervention, gain further insights into the mechanism of action of the intervention and seek recommendations for improvements.

## Methods

This qualitative study involved 15 semi-structured interviews with women within a few weeks of receiving the intervention. Participants for interview were selected purposively by the counsellor (author LS) delivering the intervention to include women from urban and rural areas and those who did or did not appear to respond to the intervention; both users and non-users of contraception. The counsellor telephoned participants to ask if they were willing to participate in the interview study.

The topic guide was developed to explore women’s experience of the intervention, aiming to identify active components of the intervention, and seek recommendations for improvements (Table 1). Questions were included on duration and frequency of messages, content of the intervention and any subsequent behaviour change. Participants were also asked about their experiences of participating in the study and will be reported elsewhere. Participants that attended for clinic interview were given $4 USD to compensate for travel expenses.

**Table 1:** Interview topic guide

**1. MOTIF mobile phone-based service**
You recently used the new Marie Stopes mobile phone post-abortion service. Could you please tell me about your experience of the service?
**a. Voice Messages (outgoing)**
I would like to hear your experience regarding the voice messages.
• Tell me what it was like when you received the VMs? • What did you think about the VMs? / How did it make you feel? • Can you remember what were you told about the VMs when you signed up?
POSSIBLE PROMPTS
i. How many voice messages did you listen to? ii. Do you recall what the voice messages said? iii. What was your understanding of the VMs? iv. What were you doing at the time you received the VMs? v. Any comments about the sound quality / voice of the message? vi. What did you expect to happen if you pressed ‘1’, ‘2’ or ‘3’? vii. Did you respond to the VM?
1. If so, what number did you press, and why?
viii. Did anyone else listen to the VM instead of you?
1. Any consequences of this?
**b. Pill/injection reminders (if relevant)**
i. Did you receive any pill or injection reminder VMs?
1. If so, what was your experience of this?
**c. IVR system and leaving messages (incoming)**
i. Did you ever call into the service?
1. If so, what was your experience of this?
**d. Counselling**
• Did you receive any direct phone calls from the counsellor?
If yes,
• What was it was like when you spoke to the counsellor? • What did you think about speaking to the counsellor? / How did it make you feel?
POSSIBLE PROMPTS
i. What kind of information were you given from the counsellor? ii. What support do you receive about post-abortion care? (E.g. medical, emotional) iii. What information were you given about contraception? iv. What did you think about any advice you were offered about contraception? Any conflict with clinic service provider advice? v. Any counsellor contact with husband/partner, any links with model clients vi. Where do you think the counsellor is based? Do you think she is old or young? Would like to see her picture before participating in the service?
**2. Support/change in behaviour/unintended consequences from intervention**
• In general, (thinking about VMs and counselling), did anything happen to you as a consequence of receiving the service (positive of negative)? OR did you do anything different? • How would this compare to if you hadn’t received the service?
POSSIBLE PROMPTS
i. Any support with SE’s/ to continue using method? ii. Any support starting or switching to a new method iii. Clinic attendances for check up’s/post-abortion follow up appointment avoided? Any time/money saved? iv. Use other services in addition to MSI? Why? v. Any suggestions for the service?
Would anything else have worked better for you? Would you recommend it to a friend seeking abortion services?
POSSIBLE PROMPTS
• Length of service, voice messages versus direct calls etc. • How could we make it more likely for you to listen and respond to VM? • Would you be prepared to pay an additional fee for the service? If so, how much?
**3. Views on trial**
• What was your experience of participating in the trial?
POSSIBLE PROMPTS:
a. Any comments on the recruitment process (information for participants, consent)? b. Any comments on phone follow up? c. Any comments on reimbursement to cover time/costs for interviews? d. Any suggestions for improvement?
Can I finally ask you for any final comments that have not been covered in this interview?

*VM* voice message, *SE* side-effects

Interviews were conducted between 30 October and 23 November 2013. Author CS conducted six interviews (four at the clinic, and two at clients houses) with author UV interpreting, author RW conducted three interviews (at the clinic) with UV interpreting, and UV conducted six interviews (four at the clinic, and two by phone). Participants were provided with an information sheet to read, or it was read to them, and provided signed or thumb-printed consent, or recorded verbal consent for the phone interviews. Interviews were recorded and transcribed by Cambodian research assistants (medical students) to English. NVivo 11 software was used to store and code all transcripts [13].

We undertook a simple thematic analysis [14]. This involved familiarization with the interview transcripts, identification of key themes, coding the data according to appropriate thematic references, comparison of themes across and within cases. CS and CF read the transcripts to identify key themes. CS coded all the transcripts and compared themes across and within cases. CF and UV coded some transcripts. Key themes are supported with quotations which have not been edited apart from obvious typos to avoid unintentionally changing the meaning. Ethical approval was obtained from ethics committees at the London School of Hygiene and Tropical Medicine and Marie Stopes International and the Cambodia Human Research ethics committee.

## Results

The characteristics of the 15 interview participants are shown in Table 2. Participants’ age ranged from 22 to 41. Most women were married and employed, but we also interviewed a student and an entertainment worker. Twelve women were using a contraception method at the time of interview, eight of whom were using long-acting methods. We identified themes relating to communication via mobile phone, supporting contraception use, broader post-abortion care, interaction with family and friends and suggestions for improvement.

**Table 2:** Characteristics of interview participants

No.	Age	Occupation	Marital status	Residence	Post-abortion contraception use
1	22	Housewife	Married or living together	Urban	Used oral contraceptive post-abortion
2	31	Employed	Married or living together	Urban	Didn’t use PAFP then had IUD inserted after repeat abortion
3	30	Self-employed	Married or living together	Urban	Returned to clinic for IUD insertion
4	24	Housewife	Married or living together	Rural	Used oral contraceptive post-abortion but discontinued as husband working away. Sent oral contraceptive reminder message
5	33	Housewife	Married or living together	Urban	Used IUD post-abortion
6	34	Self-employed	Married or living together	Rural	Returned to clinic for implant insertion
7	34	Self-employed	Married or living together	Urban	Returned to clinic for IUD insertion
8	34	Factory worker	Married or living together	Rural	Used oral contraceptive post-abortion but discontinued as husband living away
9	25	Student	Married or living together	Rural	Advised to have abortion for medical reasons and avoid pregnancy for a year; using oral contraceptive. Sent oral contraceptive reminder message
10	25	Factory worker	Married or living together	Rural	Returned to clinic for IUD insertion
11	22	Student	Married or living together	Urban	Using oral contraceptive. Sent oral contraceptive reminder message
12	20	Student	Never married or living together	Urban	Using oral contraceptive
13	38	Self-employed	Married or living together	Urban	Used oral contraceptive post-abortion then had IUD inserted
14	41	Farmer	Married or living together	Rural	Used implant post-abortion
15	25	Entertainment worker	Never married or living together	Rural	Not using contraception as not in relationship

### Communication by mobile phone

Most women reported that communication via mobile phone was convenient. Women reported listening to the voice message if it was received at a convenient time and thought the message was easy to understand. Whilst most women had forgotten the content of the voice message, most recalled the concept of pressing a number on their keypad and reported that it was good to have the option to request to speak to a counsellor or not. Women reported pressing ‘1’ if they had a question or health problem or pressing ‘2’ or ending the call if they were busy, had no problems, or did not want to disturb the counsellors.

*“Sending voice message wasn’t the disturbance because if we wanted to talk to her, we just talked and if we didn’t want to talk, we just pressed number 2 or 3 if we didn’t want her to call us, she would stop calling us” (interview 11)*

*“I think that pressing number 1 or number 2 is better because when I face problem, I just press number 1 or number 2… That’s why it is quite important for me” (interview 9)*

Several women reported that receiving messages or speaking to the counsellor was a convenient way to obtain support for health issues or remind them about contraception and saved the time and costs of clinic attendance.

*“These messages help a lot-especially for those who live far away from here like me. Because of busy, I might forget to use contraception methods, but when I listen to voice messages, I can remember. If do not want to have pregnant, I just listen to it. Voice messages always remind me…Whenever I listen to voice messages, I feel like someone stays next to me and supports me about using contraceptive methods” (interview 9)*

*“Counseling via phone call gives advantage…because we don’t have to come to PET [health worker], spend money to clinic directly, and we discuss with her and if we don’t discuss with her and we have to come to PET, we spend money first for travel fee and second for PET fee” (interview 6)*

Conversely, a few women reported missing calls if they were away from their phone. Two women reported that messages could be intrusive if received at an inconvenient time e.g. when busy at work.

*“The message just tell me to click one or two for answer… at that time I still work but if I’m busy, it’s ok but sometimes when I’m very busy and like stress and sometimes it’s annoying” (interview 2)*

### Supporting contraception use

Many women reported receiving information and increasing their knowledge on a range of contraceptive methods. Several women reported that the counsellor would ask if they were using a method, and the phone call provided an opportunity for women to ask questions about contraception.

*“She explained even about drug, IUD/implant, injection, oral pill, and IUD, and condom. She explained all. She told me a lot” (interview 5)*

*“The message ended and a short while, she would call me back… I asked her that, for example, we forgot to take the pill… What should I do if [I] forgot taking till 3 days?” (interview 11)*

Some women reported that they decided to adopt a long-acting method of contraception after speaking to the counsellor. A few women described being given information on discounts or where to access services.

*“After I got information from counsellors I then went to clinic to insert IUD…I learnt how to insert IUD and taking pills. Inserting of IUD is much more easier. Talking pill has to be on time and take it daily but for IUD we do not have to do like this. We can have sex whenever! Counselling service made me feel confident because I thought that medical science is better than our thought. Some people said that IUD can move around, but when I came to ask counsellor, she said that IUD did not have legs to move around…. I also told people I know to insert IUD as well” (interview 10)*

*“She explained me to have contraception here, use implant at this clinic, there was discount” (interview 6)*

Several women reported that the counsellor provided advice if they were experiencing side-effects from contraception; either advising the women to attend the clinic for examination or providing reassurance which led to the women continuing to use the method.

*“She said that the side effects of IUD lasted 3 months, I remember that. And after these 3 months, our body can tolerate with it, it will be alright…and now I am alright” (interview 3)*

Three women received a specific pill reminder message at one-month, but only one woman recalled receiving this. However, several women using oral contraceptive reported that the counsellor had emphasized the importance of taking the pill regularly and the risk of consequent pregnancy, resulting in a change in behaviour.

*“I was confident because she said that when we took the pill, we had to take regularly. If we didn’t take the pill regularly, it was possible to be pregnant… so I changed my habit according to her” (interview 1)*

### Broader post-abortion care

An important aspect of the intervention was the sense of care and emotional support provided as well as support for specific problems. Many women reported feeling happy because somebody was taking care of them, or asked about their health or more generally about what they were doing. Several women reported that the counsellor provided encouragement and that they gained confidence and felt less afraid.

*“For that voice message that I received, I think that it’s good. It’s good and like makes me feel warm like there’s someone take care about us” (interview 8)*

*“When I received that voice message, I felt that… she encouraged me and she loved our health so that she wanted to know about our health if we were healthy” (interview 12)*

One woman reported receiving support for suicidal feelings.

*“Struggle in my life, because sometimes I want to commit suicide, but counsellor not allow me to do this, they encourage me to be strong” (interview 15)*

The counsellor provided support for post-abortion health concerns such as abdominal pain, vaginal bleeding or discharge. In some cases the counsellor would provide reassurance or otherwise suggest the women attend the clinic for a check up.

*“After I used medicine I asked her my problem “why around my abdominal still painful? Why I cannot do something? Even just walking, I cannot walk”. Counselor said that bleeding it just side effect of medicine, but if you has much bleeding you need to go back to clinic. I followed up myself and I found that there was just small bleeding, so I decided not coming to clinic” (interview 9)*

A few women reported gaining general information about illnesses and health promotion.

*“If I make comparison, I see a lot of changing. Before I seldom to hear information, by the time I have started to use services here, I can consult at anytime and I know more about illness problem that often happen to women and its prevention” (interview 9)*

### Interaction with family and friends

In most cases women listened to the voice messages on their own. Some women reported that they were able to discuss topics in confidence that they couldn’t discuss elsewhere or with others, for example, at a hospital or with family members.

*“The reason why I believe her because she encouraged me that she wouldn’t tell other about my secret and what she talked with me she kept in secret…I believe that they can help a lot of other women, sister, because woman is shy, and sometimes she can’t talk to other… it just sometimes we felt embarrassed. There’re some matters that we don’t want other to know” (interview 12)*

A few women reported that they would hang up, or arrange to speak to the counsellor at a different time if messages were received or the counsellor called in the presence of others.

*“Sometimes there was my mother; I didn’t want to talk because I was shy and I didn’t want her to know. But if I stayed with my family, my couple, there was no problem. But sometimes…he didn’t stay with me every time, but we didn’t want like questioning words like I was sick like this or like that I didn’t want to talk in front of my mother, and my younger siblings. But she called me when I was alone or with my family, I would talk and there was no problem” (interview 8)*

If few women reported that family members or friends might ask questions after listening to a voice message in their presence, but husbands’ were generally supportive.

*“No one listens to my voice messages because my phone stays with me all the time. Even my husband, he also does not hack and listen alone. When new message coming, my husband told me… Moreover my husband always asks me questions when I finished listen to messages because he concerns much about my health condition” (interview 9)*

In other cases, women reported deliberately sharing intervention content. One woman reported using the speakerphone so that others could listen to the message. There were a few instances where women reported having subsequent conversations with friends or family members to recommend Marie Stopes services or contraception methods.

*“I never let anyone listen to it but I brought my younger sister who wanted to use IUD like me. I always told her that I felt well” (interview 3)*

There were a few instances where someone else listened to the message or spoke to the counsellor because the phone had been shared with another family member but there were no reported instances of harm as a consequence.

### Suggestions for improvement

All of those interviewed thought that the service should be offered to women in the future. One participant suggested focusing on less educated women.

*“I do not have any request because it is good enough already. I rarely to see services like this in others organization” (interview 9)*

Most women were positive about voice messages, although a few women expressed a preference for direct phone calls or text message. Although most women couldn’t remember how frequently they received messages, most reported that a frequency of two times a month was enough.

*“I want direct phone call… I am not interested in voice message at all because it takes long time, but if you call me, I once pick up the phone call” (interview 1)*

*“Text message. Sometimes when I go bathroom when comeback, I can see message rather than missed call.” (interview 15)*

Most women thought that the intervention duration of three-months was sufficient and that the messages were no longer required e.g. *“not really necessary for me because I do not have any problem now” (interview 14).* However, a few women reported that they would have liked the voice messages to continue beyond 3 months in case they experienced problems in the future.

*“There was a message said that this message was the last message. To me, when the message said that it was the last message, I felt regret and I didn’t want” (interview 3)*

*“Sometimes I don’t know in the future I will meet what problem, sister, so I am difficult to call her. When she sends voice message to me, she has my number and that number is easy for me to call to ask her too” (interview 12)*

Most women reported that they would be happy to pay a small fee (e.g. $1-2USD) for such a service, but a few thought it should remain free of charge. A few women suggested that the messages support other health topics.

*“We go to small pharmacies, we have to spend the money too; therefore, I don’t mind about paying this amount. This is for our health too” (interview 3)*

*“I am interested in general health short voice messages if we send you weekly? For example, messages tell you how to prevent from diseases, how prevent from pregnancy, STI and so on…I want these services still providing for long period” (interview 14)*

## Discussion

### Summary of main results

The majority of women were positive about the mobile phone-based intervention to support contraception use and reported it to be convenient in a number of ways.

Most women liked being able to respond to the voice message to request to speak to a counsellor or not. However a few women found the voice messages intrusive. Phone counselling was a convenient way to ask questions or get advice without going to a health centre. Women reported that the intervention, in particular phone counselling, supported contraception use by provision of information, encouragement, reminders to return to clinic, reassurance and advice for problems and had a positive effect on contraceptive uptake and continuation. Women reported a sense of being cared for and received support for additional physical and emotional issues. Counselling allowed women to discuss issues with the counsellor in confidence, although in some cases the intervention content was shared with others, either deliberately or unintentionally. Most women thought that the duration of the intervention and frequency of messages were acceptable.

### Strengths & limitations

This study provides insights to the intervention gained by in-depth interviews with women. A strength of our methodology is that most of the interviews were conducted by female researchers, which is considered more appropriate for reproductive health research in order to minimise ‘social distance’ between researchers and subjects [15].

Our study also has some limitations. It is possible that women would have been more likely to agree to be interviewed if they had had a positive experience of the intervention. We did not document if any women refused to participate. Most of the women interviewed were using a contraceptive method and hence we were unable to assess differences in accounts between contraception users and nonusers. As in the trial, most of the women interviewed were married, and single women and entertainment workers were under-represented [5]. The interviews may have been prone to social desirability bias, particularly as some were conducted by western researchers, although it is encouraging that a range of views were expressed. Although small numbers of women were interviewed, few new ideas resulted from the later interviews, and a degree of ‘saturation’ was reached.

Another potential limitation is that it wasn’t always clear if the woman was referring to voice messages or counselling when analysing the interviews. Hence it was not always possible to attribute women’s reports to the automated or counsellor-delivered components of the intervention. As a consequence of these potential biases, it is not possible to conclude that all relevant themes were identified.

### Interpretation & comparison with existing literature

To our knowledge, this is the first in-depth interview study reporting participants’ perceptions of a mobile phone-based intervention to support contraception use alongside a randomised controlled trial, although participants were asked questions regarding their satisfaction in two RCTs of oral contraceptive adherence interventions in the USA [2, 3]. Our finding that participants were positive about the intervention is consistent with previous studies assessing participants experience of mobile phone-based interventions in other areas (e.g. HIV medication adherence, maternal and child health, sexual health, smoking cessation) [2, 3, 16–19].

Communication via mobile phone was a convenient way to discuss contraception or health issues, saving money and time in comparison to going to a health centre, as previously reported [16]. Our previous analysis found that the proportion of women that requested to speak to a counsellor (pressing ‘1’) decreased from message one to six [12], which is consistent with reports from the interviews that health issues often resolved over time.

Our finding that messages could be inconvenient and intrusive for some women highlights the limitation of real-time voice messages as a delivery mechanism, contrasting with other studies of interventions delivered by mobile phone where participants could check messages at their convenience [18, 20].

It is unclear if the voice messages improved contraceptive use. Participants in other trials reported that daily educational text messages helped them remember to take oral contraceptive or HIV medication [2, 3, 16]. However, reviews of trials of text messages for medication adherence show limited evidence [1, 21]. In our intervention, the reminder to use contraception appeared to be related to providing general motivation rather than a daily prompt.

Interview findings suggest that the intervention included components identified as best practices for contraception counselling including developing close personal relationships, building trust, and adequate counselling regarding side-effects [22], which may not have been possible with a fully automated intervention. It is not clear to what degree provision of information about discounted services influenced uptake as clients still had to pay a user fee and incur associated travel and opportunity costs. All women would have received contraception counselling at the time of seeking abortion services, as per current recommendations [23]. Findings from this study indicate some benefits to providing on-going support for women to reconsider their contraceptive options and provide support for side-effects as they arise.

The intervention provided additional benefits that were broader than the trial definition of success (i.e. contraception use). Our findings resonate with other qualitative studies of mHealth interventions that reported a feeling that someone cares [16, 19]. The action of sending messages (‘push’) to participants may contribute to this feeling, as previously reported [18, 24].

Our finding that women received support for management of physical and emotional health issues is consistent with studies elsewhere; post-abortion mobile phone follow up was demonstrated to reduce women’s anxiety and stress in South Africa [25], and be acceptable and preferable to a clinic visit in the UK [26].

Although there were no reports of any adverse advents as a result of others listening to messages, our interview participants were mostly married and more likely to have disclosed having had an abortion to others. Women concerned about others listening to messages may have elected not to receive the intervention. Phone sharing is common in Cambodia and possible unintended consequences should be considered when developing future intervention content [27].

Other evaluations of text message interventions for contraception and sexual health have found that participants would re-read messages which might lead to conversations with family or friends [18, 28]. Although this was not possible with our voice messages, some women reported recommending long-acting contraception or Marie Stopes services to other people. This additional contraception use was not captured with the trial follow up but could be evaluated in further studies.

### Mechanism of action

This study provides some insight into the mechanism by which the intervention resulted in behaviour change. The voice messages appeared to act as a conduit for additional support rather than directly influencing behaviour change, but may have promoted engagement in the intervention, as previously reported [29].

The majority of support for contraception uptake and continuation and other post-abortion issues appeared to be provided by the counsellor calls rather than the voice message. The counselling addressed *intrapersonal* determinants of contraceptive use, in particular health concerns, as well as *capability*, *motivation* and *opportunity* to use contraception, as per our conceptual framework [30–32]. The relatively intensive intervention whereby the counsellor could develop a relationship with the woman and deliver personalised support over a short duration likely influenced uptake of long-acting methods but was less effective for continued adherence to short-acting methods [5].

The intervention included five behaviour change techniques; two could be attributed to the voice messages (*provide instruction*, and *prompt practice)* and three to the counselling (*provide information about behaviour-health link*, *provide information on consequences*, *prompt barrier identification* [1, 33]*.* Findings from this study suggest that another component of the counselling was to *provide general encouragement*. In general the wider literature suggests that multi-faceted, more complex interventions are more likely to be effective [20, 21, 34].

### Improving the intervention

Most women thought the service should be offered to women in the future with few suggestions for improvement. There was no evidence that the intervention effect varied by level of education [12]. Some women expressed a preference for direct phone calls or text message but this appeared to be due to timing of the message and concern about missing the call. Most women thought that the frequency of messages and duration of the intervention were acceptable, although as with previous trials of oral contraceptive reminders, some would have liked the intervention to continue [2, 3]; perhaps more as a ‘safety net’ to support general health.

Our intervention increased effective contraception use at four-months, soon after the intervention had ended, but an effect was not demonstrated at 12 months partly due to the trial not being powered for the outcome at 12 months and increased attrition at 12 months. A previous trial of text message reminders for OC adherence found the intervention effect was greater whilst the intervention was on-going rather than after the intervention ended [2]. This raises the question of whether a longer intervention would have resulted in a more sustained effect. However, evidence that interventions encouraging medication adherence are more effective for short-term rather than long-term treatments suggests only modest improvements might be expected [20].

### Implications for practice/research

Findings from this analysis have some implications for practice and future research. Our findings suggest there may be benefits from providing this intervention (such as increased use of long-acting methods) but the long-term effects require further evaluation. Future interventions for post-abortion contraception could consider including messages to support comprehensive post-abortion care more broadly and should anticipate that women may have a range of issues and be prepared to manage these safely. The intervention could be adapted for use on smart phones and could utilise password protected voice or text messages that are retained on the participants phone. This might improve the response to messages and facilitate sharing of intervention content with others. We recommend that mobile phone-based interventions for on-going support for PAFP should be integrated with counselling at the time of seeking services. Operational research could examine clients’ actual willingness to pay for such interventions, and the effect of varying the duration of the intervention. Further adequately powered trials of mobile phone-based interventions to support contraception use are needed.

## Conclusions

The majority of women were positive about the mobile phone-based intervention which provided support for contraception use as well as additional physical and emotional issues. The study provides some insights into the possible mechanism of action and considers how the intervention could be improved.
